# A comprehensive bibliometric exploration of hydrogel applications in spinal cord injury

**DOI:** 10.3389/fphar.2025.1606186

**Published:** 2025-08-28

**Authors:** Guosong Han, Huihui Zhang, Zhixiang Li

**Affiliations:** ^1^ Department of Orthopedics, The Third Affiliated Hospital of Anhui Medical University (The First People’s Hospital of Hefei), Hefei, Anhui, China; ^2^ Oncology Department of Integrated Traditional Chinese and Western Medicine, The First Affiliated Hospital of Anhui Medical University, Hefei, China

**Keywords:** spinal cord injury, biomaterials, hydrogel, bibliometric analysis, hotspots

## Abstract

**Introduction:**

Spinal cord injury (SCI) represents a severe traumatic disorder of the central nervous system, leading to potential loss of motor and sensory functions. Its intricate pathological mechanism renders its treatment a formidable challenge. Recently, hydrogels have emerged as promising materials for spinal cord repair due to their exceptional biocompatibility and biodegradability, garnering significant attention. Consequently, extensive research on hydrogel applications in spinal cord injuries aims to provide an in-depth understanding of this field’s current state and delineate future research trajectories.

**Methods:**

A thorough search was conducted using the Web of Science Core Collection (WoSCC). Bibliometric tools such as CiteSpace, VOSviewer, Scimago Graphica, R and Bibliometrix software were employed to construct a knowledge map regarding the application of hydrogel in SCI.

**Results:**

A bibliometric analysis of 1,015 publications between 2000 and 2025 elucidates the current research landscape, developmental trends, academic impact, and emerging knowledge dissemination patterns in hydrogel applications for SCI. The international collaboration in hydrogels-based SCI research exhibits a China-U.S.-centered network structure: as the top two publishing countries (464 vs. 278 publications), they maintain the closest bilateral collaboration, collectively forming a prominent transnational research network. The journal Biomaterials boasts the highest number of publications with 58 articles. Among prolific authors, Shoichet, Molly S., has authored the most papers, totaling 38 articles. There is a notable collaboration among various countries and institutions, with current research predominantly focusing on inflammation, apoptosis, nanoparticles, and injectable hydrogels. These efforts aim to achieve functionalized hydrogel regulation of microenvironmental changes, emerging as a focal point in contemporary research. This research highlights the latest trend of hydrogels in the treatment of SCI, thus pointing out the direction for new treatment strategies.

**Discussion:**

The current research focus, which include the integration of functionalized hydrogels with biological factors, are setting the stage for subsequent investigative endeavors and the eventual clinical application of hydrogel in the treatment of SCI. This comprehensive analysis not only delineates the current state and emerging frontiers of hydrogel-based treatments for SCI but also provides a roadmap for future innovation.

## 1 Introduction

Spinal cord injury (SCI) irrevocably results in profound sensory and motor dysfunction below the level of injury, often leading to persistent paralysis (Cowan et al., 2020; Jiang et al., 2019; Liu et al., 2024a). It is now widely acknowledged that the hostile environment postinjury poses a significant impediment to successful neuro-regeneration and functional recovery (Ahuja et al., 2017). To date, there is no efficacious clinical treatment for nerve regeneration and functional restoration following SCI. Consequently, SCI is considered one of the most intricate and challenging neurological disorders encountered in clinical practice. The pathophysiology of SCI is multifaceted and comprises two distinct stages: primary injury and secondary injury. Primary injury represents the initial mechanical trauma to the spinal cord tissue. Secondary injury, on the other hand, encompasses a series of maladaptive responses to the initial insult, such as apoptosis, lipid peroxidation, and inflammation, collectively creating a hostile inflammatory milieu (Ahuja and Fehlings, 2016; Silva et al., 2014; Wang et al., 2019). This milieu, coupled with the inherently limited capacity for nerve regeneration, culminates in the formation of a dense neuroglial scar, which serves as a formidable barrier to axonal regrowth (Park et al., 2018). These factors coalesce to undermine the central nervous system’s intrinsic ability to regenerate after SCI. Currently, there are two principal therapeutic strategies aimed at spinal cord repair: modulating the inflammatory milieu and fostering nerve regeneration (Liu et al., 2023; Wu et al., 2023; Wang et al., 2024; Ye et al., 2024; Song et al., 2024; Shi et al., 2024). These approaches seek to shield the spinal cord, neutralize inhibitory factors, and stimulate axonal elongation. Hormonal treatments are routinely administered clinically post-SCI to mitigate the stress response and inflammatory sequelae, thereby safeguarding compromised neurons and forestalling further deterioration of the microenvironment. However, the efficacy of this intervention in promoting axonal and neural regeneration is limited, and excessive hormonal dosing may precipitate severe adverse effects. Conventional post-spinal cord injury treatments predominantly involve pharmacologic, physical, or surgical interventions. Surgical methods primarily alleviate compression of foreign bodies on nerves, while pharmacological treatments aim to reduce inflammation and stimulate the secretion of nerve growth factor, among other mechanisms. In contrast, hydrogels offer a three-dimensional framework that facilitates nerve cell growth and axonal extension, guiding nerve fibers towards forming new connections at the injury site. Despite the benefits of conventional treatments, achieving full functional recovery in severe nerve injuries remains challenging. Scaffold hydrogel can emulate the microenvironment of nerve tissue, providing structural support for synapse formation and enhancing the prospects of functional restoration. Engineering biocompatible scaffolds presents a novel therapeutic approach for nerve injury repair, potentially leading to more efficacious functional recovery and minimizing treatment-related complications and side effects. In recent years, innovative experimental methodologies, including cellular transplantation and the deployment of scaffolding hydrogels, have emerged to facilitate axonal elongation in the context of SCI repair, including cellular transplantation and the deployment of scaffolding hydrogels (Zheng and Tuszynski, 2023; Hu et al., 2023; Ribeiro et al., 2023). Despite the considerable promise exhibited by cellular transplantation in clinical investigations, challenges such as unpredictable cell differentiation, suboptimal survival rates, and ethical quandaries persist. With advancements in regenerative medicine techniques, an innovative strategy centred on the concept of neuronal relay formation has been proposed for the treatment of SCI. This approach leverages residual neurons to reconstruct neural networks, facilitate the transmission of neural information along spinal nerve fibres, reestablish neural circuits, and ultimately restore motor function post-SCI (Lai et al., 2021).

The establishment of neuronal repair networks necessitates the utilization of seed cells. Cell transplantation has demonstrated experimental success in clinical settings, but neural stem cells (NSCs), whether endogenous or exogenous, tend to differentiate into astrocytes rather than into neurons within an inflammatory microenvironment. Consequently, there is a pressing need to optimize the microenvironment to enhance neuronal transformation. However, direct injection of seed cells and cytokines into the site of injury frequently results in cell loss and death. Therefore, an appropriate medium is essential as a carrier to create a supportive microenvironment for the physiological activities of seed cells (Fan et al., 2018). Considering the magnitude of these problems, cost-effective and cell-free biomaterial implants are highly desirable. The use of scaffold hydrogel therapies with adjustable moduli, topologies, and patterned surfaces has been suggested as a strategy to augment neural tissue regeneration. These three-dimensional (3D) matrices possess desirable biological, chemical, and physical properties that encourage cell adhesion, growth, and differentiation (Hong et al., 2017; Koffler et al., 2019).

With advancements in tissue engineering, hydrogels have been increasingly utilized. Resembling natural soft tissues, hydrogels possess a highly porous structure conducive to cell infiltration, nutrient transfer, and signalling (Liu et al., 2024b). These materials offer microenvironmental support akin to the native state, underscoring their potential in regenerative medicine. Hydrogel scaffolds, as efficacious vehicles for stem cell delivery, not only augment the retention of neural stem cells post-transplantation but also mimic the extracellular matrix (ECM) of neural tissues by providing niches conducive to cellular residence, migration, proliferation, and differentiation (Ghane et al., 2020). The adjustable physical attributes, controllable degradability, and capacity to stabilize labile compounds make hydrogels ideal for creating supportive microenvironments around NSCs. Furthermore, hydrogels can interact physico-chemically with encapsulated drugs to modulate their release kinetics (Lu et al., 2017). Fang et al. engineered a porous hydrogel microneedle patch that sustained the release of exosomes from bone marrow mesenchymal stem cells embedded within. This innovation reduced the formation of cavernous and fibrotic tissues, promoted angiogenesis, and enhanced the survival of neighbouring tissues and axons, thereby aiding in SCI recovery and offering a continuous exosome delivery platform (Fang et al., 2023). In another study, He et al. fabricated a composite scaffold integrating decellularized spinal cord ECM gel with a hydrogel, encapsulated stem cells within, and implanted it into the injury site. This approach ameliorated motor function postinjury, dampened the inflammatory response, encouraged neuronal differentiation, and inhibited the proliferation of reactive astrocytes, suggesting that this approach is a promising therapeutic avenue for treating spinal cord injuries (He et al., 2022). Despite the significant potential of hydrogel-based SCI repair, the field lacks systematic quantitative analysis of research dynamics, collaborative networks, and cutting-edge directions, hindering comprehensive understanding of developmental trajectories and breakthrough opportunities. This study employs bibliometric analysis of 1,015 articles (2000–2025) to identify publication trends, characterize collaborative networks among contributing countries, institutions, and authors, and explore evolutionary paths of hot keywords, Frontier directions, and academic contributions of high-impact journals and seminal studies. Through systematic synthesis and visual analytics, we aim to reveal current research status and gaps, provide data support for interdisciplinary collaborations spanning materials science, neurobiology, and clinical medicine, and offer theoretical references for clinical translation of hydrogel therapies. A comprehensive literature exposition and analysis in this domain are essential to guide and inspire ongoing research endeavours.

Bibliometrics is an innovative analytical tool that employs statistical methods and data visualization to rapidly discern the structure and trends within a given topic or field (Zhiguo et al., 2023). This approach facilitates the identification of pertinent nodes and the extraction of valuable insights from vast informational datasets (Chen and Song, 2019; Brandt et al., 2019). Prominent among the bibliometric software suites are CiteSpace, VOSviewer, Scimago Graphica, R and Bibliometrix (Chen, 2006; Chen, 2004; Chen et al., 2010). The analysis process yields collaborative networks encompassing authors, institutions, countries, etc.; thematic co-occurrence networks featuring keywords and journals; and co-citation networks. Consequently, the evolution of a field can be comprehensively traced through bibliometric analysis (Ellegaard and Wallin, 2015).

## 2 Materials and methods

### 2.1 Data collection

**FIGURE 1:**
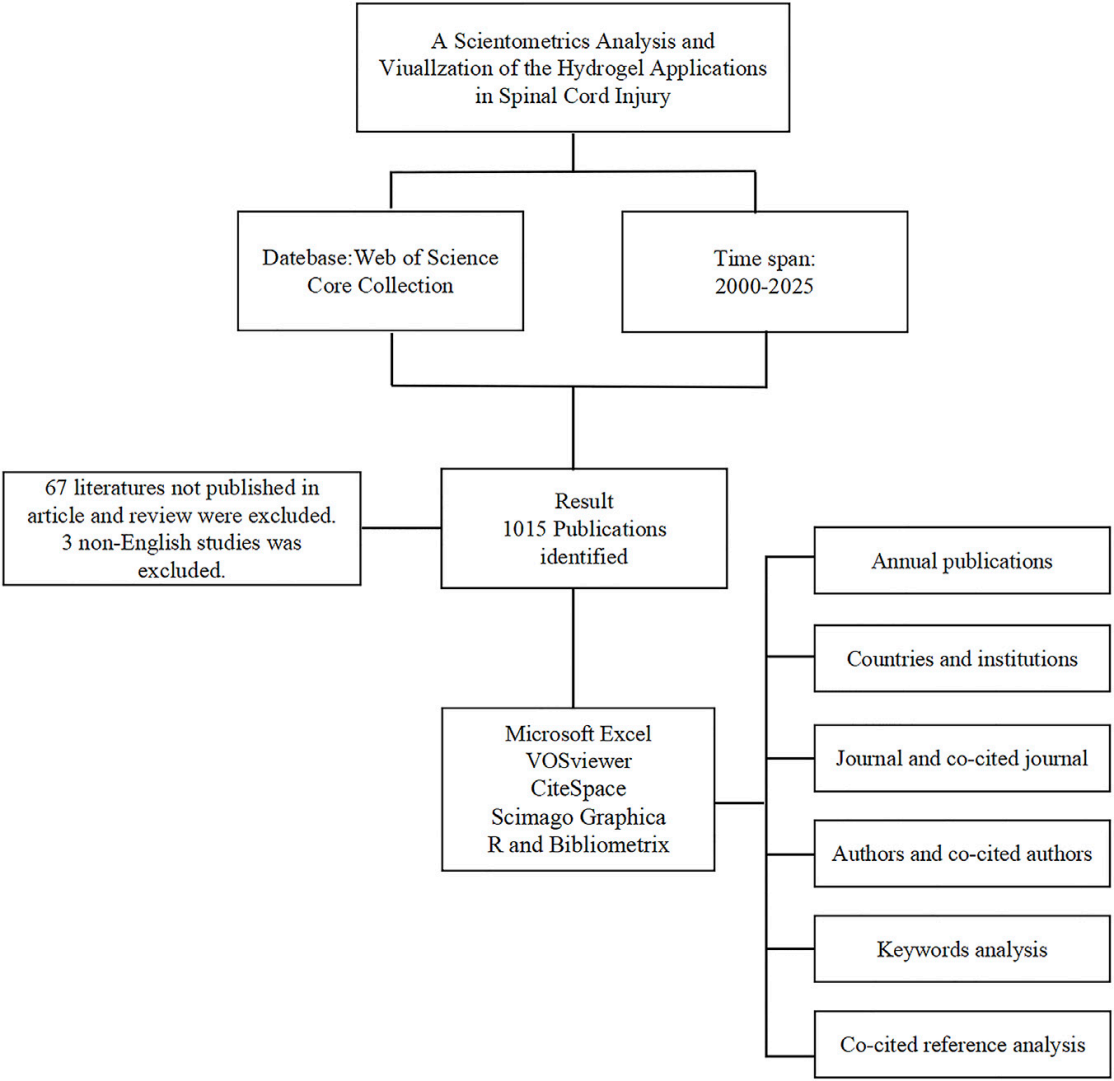
Schematic diagram of the data retrieval and analysis process.

A comprehensive literature search was performed on 01 March 2025, utilizing the WoSCC through Clarivate Analytics (Yeung, 2019). The primary research areas included spinal cord injury and hydrogels, employing a search strategy that integrated both subject terms and free-text keywords. The detailed search algorithm was as follows: TS = [(“Spinal Cord Trauma” OR “Cord Trauma, Spinal” OR “Cord Traumas, Spinal” OR “Spinal Cord Traumas” OR “Trauma, Spinal Cord” OR “Traumas, Spinal Cord” OR “Myelopathy, Traumatic” OR “Myelopathies, Traumatic” OR “Traumatic Myelopathies” OR “Traumatic Myelopathy” OR “Injuries, Spinal Cord” OR “Cord Injuries, Spinal” OR “Cord Injury, Spinal” OR “Injury, Spinal Cord” OR “Spinal Cord Injury” OR “Spinal Cord Transection” OR “Cord Transection, Spinal” OR “Cord Transections, Spinal” OR “Spinal Cord Transections” OR “Transection, Spinal Cord” OR “Transections, Spinal Cord” OR “Spinal Cord Laceration” OR “Cord Laceration, Spinal” OR “Cord Lacerations, Spinal” OR “Laceration, Spinal Cord” OR “Lacerations, Spinal Cord” OR “Spinal Cord Lacerations” OR “Post-Traumatic Myelopathy” OR “Myelopathies, Post-Traumatic” OR “Myelopathy, Post-Traumatic” OR “Post Traumatic Myelopathy” OR “Post-Traumatic Myelopathies” OR “Spinal Cord Contusion” OR “Contusion, Spinal Cord” OR “Contusions, Spinal Cord” OR “Cord Contusion, Spinal” OR “Cord Contusions, Spinal” OR “Spinal Cord Contusions”) AND TS = (“Hydrogel” OR “Hydrogels” OR “*In Situ* Hydrogels” OR “*In Situ* Hydrogel” OR “Hydrogel, *In Situ*” OR “Patterned Hydrogels” OR “Patterned Hydrogel” OR “Hydrogel, Patterned”)] AND [Language = (English)]. Inclusion criteria focused on articles and reviews, and excluded other document types, with publication years restricted to 2000–2025. Eligible literature was exported in plain text format for subsequent analysis via using CiteSpace, VOSviewer Scimago Graphica, R and Bibliometrix. Figure 1 provides a detailed illustration of the data collection and processing steps employed in this experiment.

### 2.2 Data analysis

The raw data from the Web of Science Core Collection (WoSCC) utilized in this study were processed using specialized software. CiteSpace is a Java-based bibliometric and visual analysis software developed by Chen Chaomei that was designed to uncover research trends within a specific field by extracting highly cited keywords and references, among other features. In our study, CiteSpace is mainly used for co-citation analysis, keyword co-occurrence, and reference burst detection. Burst analysis identified keywords/references with significant citation surges using the Burst Detection algorithm. The aim was to investigate the frequency of use or citation at specific time intervals. In the co-citation view, the left side signifies the discipline of the journal where the article was published, while the right side indicates the discipline of the journal where the article was cited. These nodes are linked by a curve that forms the citation path, with the thickness of the curve indicating the strength of the co-citation. The timeline view is a visual analysis method that integrates time slicing with clustering algorithms.

VOSviewer is an alternative bibliometric analysis software adept at creating and visualizing knowledge maps, effectively depicting various types of clustering, coverage, and density (van Eck and Waltman, 2017; van Eck and Waltman, 2010). VOSviewer is employed for network visualization of countries, institutions, authors, journals, and keywords. Clustering was performed using the VOS algorithm with cosine similarity, and node size reflected publication/citation counts. In our study, the visualization results of countries, institutions, authors, journals, and keywords were analysed to provide a more visual representation of these entities through the analysis of the number of inquiry targets, partnerships, or temporal nodes via node size, as well as the distribution of node clusters.

Microsoft Excel was used to analyse changes in the number of published articles, countries, and journal literature. For the geo-visualization of publishing countries, we utilized Scimago Graphica software to convert the digital information of different countries into graphs, which facilitated our observation of global participation in this field of research. It can generate a geographic map of global research results with color-coded regions to represent information such as publication volume. Metrics such as citation frequency and H-index were calculated using R and Bibliometrix to assess the impact and scholarly value of the literature.

### 2.3 Data visualization interpretation

In this study, the data visualization was systematically designed to reveal the structural patterns and temporal dynamics of hydrogel application in SCI research, employing a variety of bibliometric tools to transform the complex dataset into an interpretable visual narrative. The target literature was screened according to the subject terms and free-text keywords, and the literature data (in plain text format) was exported from Web of Science Core Collection (WoSCC), for the analysis of co-citation analysis, keyword emergence, and timeline mapping, we used the Citespace software, imported the above data into the software, and set up the conditions as time span (2000–2025), years per slice (1), selection criteria (g-index k = 25 or Top N% = 2), link retaining factor (LRF = 3), links (strength: cosine, scope: within slices), cluster labels were extracted by the log-likelihood ratio (LLR) algorithm, and others remained default. The required data were then exported and analyzed and processed according to the experimental purpose. For the analysis of collaborative networks, keyword co-occurrence, keyword and journal clustering, we imported the data exported from WOSCC into VOSviewer software, chose “Create a map based on bibliographic data”, followed by setting the conditions according to the experimental needs, we get the required results. For the analysis of countries, we need to do a unified proofreading of the country affiliation, and keep the default value for the rest of the analysis. The geo-visualization results are obtained by processing the literature data exported by WOSCC through the VOSviewer software to generate a file in GML format. For the presentation of author influence mapping, keyword time series, etc., we used R (Bibliometrix) for analysis. The analysis website was opened using the designed program, the exported data was compressed into a zip file, and the target results were analyzed when imported into that webpage.

## 3 Results

The study included an analysis of 1,015 papers authored by 5,285 individuals from 1,100 institutions across 53 countries. These papers were published in 301 distinct journals and referenced a total of 42,405 citations sourced from 4,909 different journals.

### 3.1 Annual volume of publications and linkages between countries and institutions

**FIGURE 2:**
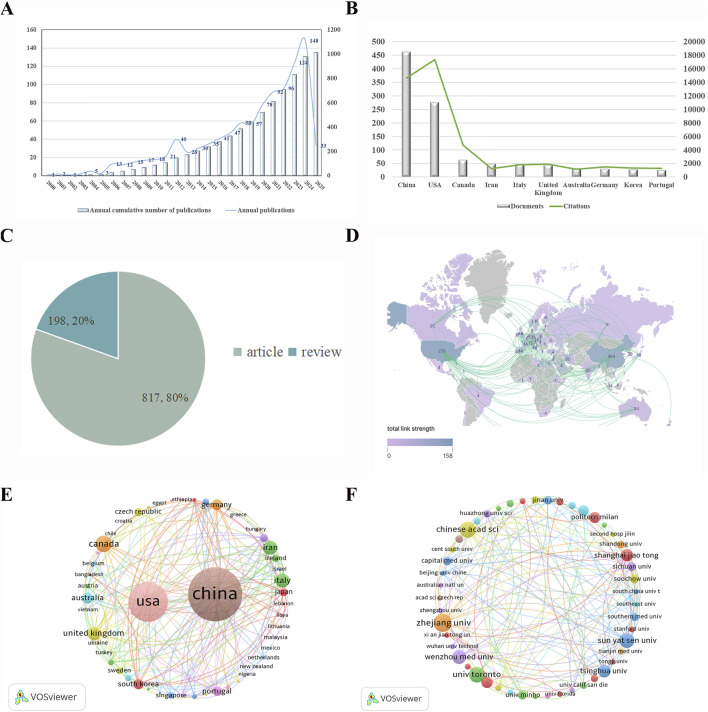
**(A)** Trend graph of the number of articles published per year in the field. **(B)** Number of articles published in each country. **(C)** Percentage of types of articles published. **(D)** Networks of global regions interacting in research collaboration. **(E)** Network map of countries involved in research related to the treatment of SCI with hydrogels. **(F)** Network map of institutions involved in research related to the treatment of SCI with hydrogels.

We systematically analyzed and organized the obtained data utilizing Microsoft Excel 2023 software, which enabled us to quantify the number of papers published annually and the cumulative total of publications from 2000 to 2025, subsequently plotting a trend line graph. As depicted in Figure 2A, the development of published articles over the past 2 decades demonstrates a general upward trajectory, although the period from 2000 to 2010 is characterized by relatively slow growth. This stagnation suggests that the application of hydrogel materials in spinal cord injury (SCI) research was still in its nascent stages during this time, with research being fragmented and lacking consistency. However, from 2013 to 2024, the field entered a phase of rapid growth, signaling a clear shift toward more intensive research into hydrogel applications for SCI repair. This acceleration indicates growing scholarly recognition and increasing interest in this approach, which may, in turn, foster the development of novel therapeutic concepts for SCI. The relatively low number of publications in 2025 can likely be attributed to the early nature of studies conducted during that period.

Figure 2B further illustrates the disparity between countries in terms of publication volume, with Chinese scholars leading the field with a total of 464 publications, followed by the United States with 278 and Canada with 65. The marked contrast between the leading countries indicates a dominance of China and the United States in this research area. In terms of citation impact, the United States clearly leads, with China trailing in second place. As shown in Figure 2C, a breakdown of the 1,015 relevant papers published between 2000 and 2025 reveals that 817 (80%) are original research articles, while 198 (20%) are review articles, further illustrating the prominence of primary research within the field.

A geographical analysis of publication distribution, presented in Figure 2D, reveals the global involvement in SCI hydrogel research, with color-coded regions representing the countries contributing to the literature. The publication volume is indicated by the numbers in each region, with China at the forefront (464 publications) followed by the United States (278). The curves connecting the countries signify the strength of international collaboration, with particularly strong links between China and the United States. This is further corroborated by the network visualization in Figure 2E, which displays the relative size of nodes to indicate the number of publications from each country. The dense network of connecting lines between China and the United States underscores the substantial level of collaborative effort between these two nations, reflecting a particularly robust research partnership.

From a global perspective, the publication landscape reveals an imbalanced distribution, with a select few countries contributing the majority of the research output. This trend is further evidenced by the comparison of node sizes, which highlights the concentration of research in a limited number of nations. Finally, Figure 2F visualizes the collaborative landscape within the field, with nodes representing institutions and varying in size according to their publication volume. Different colors correspond to distinct collaborative clusters, and the strength of cooperation both within and between clusters is evident in the density of connecting lines. These visualizations suggest that institutional collaboration is thriving not only within specific clusters but also across borders, fostering a dynamic and interconnected research community.

### 3.2 Journals and cited journals

**FIGURE 3:**
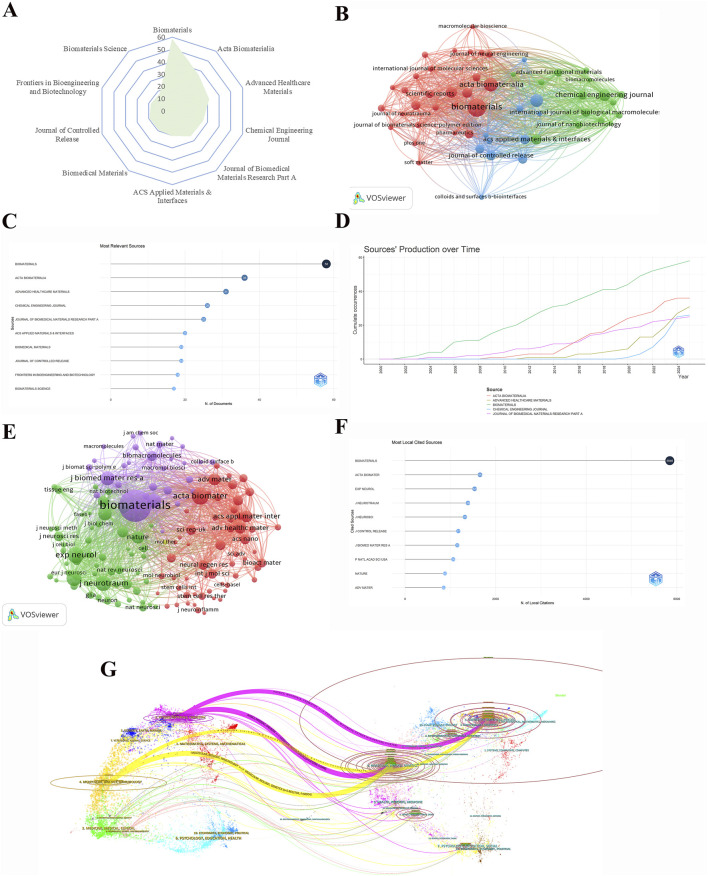
Analysis of keywords with keyword bursts. **(A)** Radar chart of the top 10 journals in publications. **(B)** Cluster diagram of journals with more than 5 publications. **(C)** Literature counts by journal in the spinal cord injury research field. **(D)** Cumulative number of occurrences of publications from various sources over time. **(E)** Cluster plot of journals with a minimal number of citations of 100. **(F)** Journals with the highest citation frequency and their corresponding number of citations. **(G)** Double map overlay of journals related to research in the field of hydrogels for spinal cord injury applications.

The information regarding journals in the field of hydrogel in spinal cord injury has been presented in tabular form using Excel, VOSviewer, R and Bibliometrix software has been utilised to visualise this information. The radar chart in Figure 3A illustrates the top ten journals with the highest number of publications in the field of hydrogel in spinal cord injury. The radar chart reveals disparities in the quantity of articles published by various journals. Specifically, biomaterials ranked first in terms of the number of articles published under this field, followed closely by Acta biomaterialia, which reflects their research heat in this field and also reveals their academic contributions to the research on spinal cord injury and hydrogel applications. Figure 3C provides a detailed representation of the specific number of articles published in these journals. The figure clearly labels the number of publications for each journal, including 58 for biomaterials which reflects the magnitude of its contribution to the field. This comprehensive dataset allows for the analysis of the publication patterns across various journals, facilitating an assessment of their respective impact and establishing them as a valuable reference point for prospective submissions and literature reviews.

The visual network graph in Figure 3B analyses journals that have published a minimum of five articles. In the graph, the size of the nodes represents the number of articles published by the journal, with larger nodes indicating greater academic activity in the field. It is evident that prominent journals such as Biomaterials and Acta Biomaterialia are particularly dominant in this field, with their numerical superiority being clearly manifested in the graph. Furthermore, the presence of connecting lines between journal nodes serves to demonstrate the inter-relationships between these publications, providing a networked perspective on the collaborative dynamics within the field. This visual network graph serves to demonstrate clearly discernible connections between journals, thereby reflecting the trend of collaborative research or similar scholarly orientations. This visualisation offers researchers a network mapping of collaborations and scholarly exchanges between journals.

Figure 3D reflects the variation in the number of publications in spinal cord injury research across journals by showing the trend of publications in journals. As demonstrated in Figure 3D, the publication volume of the biomaterials journal has exhibited a relatively stable upward trend since 2000, signifying that the journal has consistently maintained a certain academic output in this field, thereby denoting its long-term stable academic status in this domain. Conversely, the publication volume in the other journals exhibited a higher degree of stability. Prior to 2016, there was a comparatively low level of publications in this field by other journals, however, subsequent to 2016, there was a notable increase in the publications of Acta Biomaterialia, Advanced Healthcare Materials, and the Journal of Biomedical Materials Research Part A. This increase may be indicative of an escalating interest in this field.

As illustrated in Figure 3E, a comprehensive overview of the citation impact is provided by depicting journals that have received over 100 citations. In this figure, the size of the node is proportional to the number of citations received by the journal, with the biomaterials journal node being the largest, thus indicating that the journal has a high impact within the academic community. The utilisation of distinct colours in representing different citation clusters in the cited literature enables the delineation of three primary categories. The distinction in colour serves to indicate the diverse academic clusters to which journals belong. Nodes with the same colour represent journals with analogous research direction or methodology. Conversely, dissimilar colours signify disparate academic themes. Through observing the close connections between nodes, the crossover among different academic clusters is apparent, though journals within an academic cluster exhibit a high frequency of citation cooperation among them. This figure not only reveals which journals have higher citation rates in the field of hydrogel application to spinal cord injury, but also shows how researchers within this field form academic communities through citation networks.

Figure 3F provides a detailed quantitative analysis of the specific number of citations directed towards the cited journals. This quantitative display is a numerical representation of the specific number of citations for each journal in Figure 3E. The journal Biomaterials, with 5,846 citations, is the most frequently cited journal, significantly exceeding the citations of other journals such as Acta Biomaterialia, Advanced Healthcare Materials, and Journal of Biomedical Materials Research Part A. This finding indicates that articles from this journal are more influential. The analysis of this data enables a clearer understanding of the dissemination of knowledge from journals in the field of spinal cord injury. Furthermore, it provides a possible source of reference for future research.

Figure 3G depicts a map overlay of journals, illustrating the distribution of their respective topics. The published journals are positioned on the left, while the cited journals are on the right, with citation relationships represented as spline waves, predominantly in green, rose, and yellow. The two principal pathways are highlighted in green and orange. It is evident that the published journals primarily comprise categories such as Molecular/Biology/Immunology, indicated by the orange line, and Physics/Materials/Chemistry, indicated by the rose line. This alignment correlates with the research domain of biomaterials applied to spinal cord injuries. On the right side, within the cited journals, the yellow path signifies that Molecular/Biology/Immunology journals are frequently cited across research in both the Molecular/Biology/Immunology and Physics/Materials/Chemistry clusters. Additionally, the rosy red path suggests that research from Physics/Materials/Chemistry journals is often referenced in articles within the Molecular/Biology/Immunology and Physics/Materials/Chemistry categories.

### 3.3 Authors and cited authors

**FIGURE 4:**
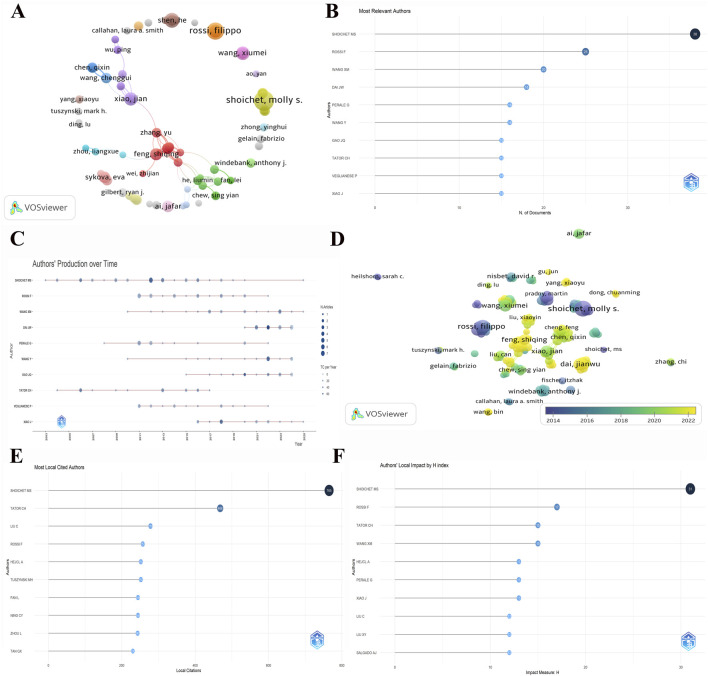
Analysis of authors and cited authors. **(A)** Cluster plot of authors with a minimum of 5 publications. **(B)** Number of documents associated with the most relevant authors. **(C)** The authors’ publication production over time related to research in the field of hydrogels for spinal cord injury applications.** (D)** Temporal clustering of authors with more than 5 publications. **(E)** Number of documents associated with the most local cited authors. **(F)** Authors’ local impact measured by H-index.

To explore the details of the publications of different authors in this field, we analysed those authors with a minimum of five publications and their collaborations using VOSviewer software. As illustrated in Figure 4A, each node in the graph denotes an author, with the size of the node indicative of the author’s publication count. Notably, Shoichet, Molly S. emerges as the author with the most publications. Figure 4B reveals that he has authored 38 articles within this domain, underscoring his excellence in the field. The next most prolific author is Rossi, Filippo, with 25 articles. Among the Chinese scholars, WANG XM and DAI JW have the second and third highest numbers of publications. The lines between these clusters indicate the collaboration between authors, thus facilitating the identification of research synergies and the establishment of collaborative connections. Feng, Shiqing and Zhang, Yu are located in the centre of the graph, connecting several research groups and forming a close collaboration network (Figure 4A). The utilisation of colour differentiation serves to highlight multiple independent research clusters. This observation suggests a propensity among some authors to pursue autonomous research endeavours. The collaborative network provides strong visual support for understanding the pulse of hydrogel development in spinal cord injury research. As demonstrated in Figure 4C, a timeline plot of authors’ publications in the field illustrates the publication activity of multiple significant authors in this research domain. The horizontal line denotes the timeframe of the authors’ publications, with the size of the circle signifying the number of articles published per year. As shown in the figure, authors such as Shoichet M.S., Rossi F., Wang XM, and Dai J.W. have continued to contribute to the field with a high frequency of publications. Shoichet M.S. and Rossi F. are the major contributors to the field, and authors such as Wang XM. and Dai J.W. have had a more notable peak in publications after 2015, which may be related to the fact that the research on the application of hydrogel in the treatment of spinal cord injury has gradually become a hot spot in recent years. In the author clustering network time diagram (Figure 4D), it is evident that the contributions of publications in this field over recent years are predominantly attributable to Chinese scholars, including Feng S.Q. and Dai J.W., among others. The increasing collaborative trend among scholars is a notable observation in the diagram. The most cited local authors in the field are presented in Figure 4E. Shoichet M.S. emerges as the most cited author, amassing an impressive 765 local citations, underscoring his excellence in the field. It is notable that other authors, including Tator C.H., Liu C., and Rossi F., also exhibit high local citation counts, suggesting that their research has exerted a substantial academic influence within the field. The H-index was utilised as a metric to assess the academic impact of the authors in this collection of literature (Figure 4F), with Shoichet M.S. demonstrating an H-index of 31. A perusal of the results presented in Figure 4C illuminates the fact that Shoichet M.S. possesses an extensive research background. This finding indicates a high academic impact within the domain of hydrogel application for spinal cord injury treatment. Rossi F. follows with an H-index of 17, which demonstrates the significant contribution of his research to the field.

### 3.4 Reference and cited reference

**FIGURE 5:**
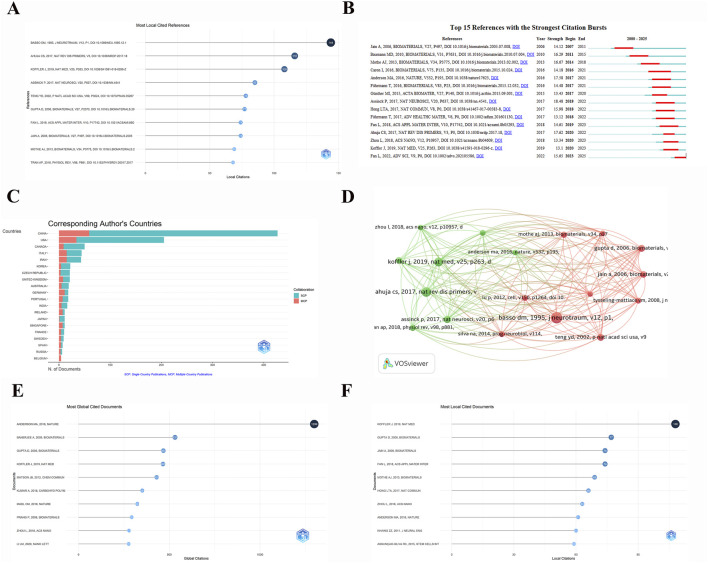
Analysis of references and cited reference. **(A)** Number of the most local cited references. **(B)** Top 15 References with the Strongest Citation Bursts. **(C)** The distribution of publications from corresponding authors by country. **(D)** Cluster graph of cited references with more than 60 citations. **(E)** Number of most global cited documents. **(F)** Number of most local cited documents.

The citation of references within the field was then explored. As demonstrated in Figure 5A, the article published by (Basso et al., 1995) in J NEUROTRAUM was the most cited, with 144 citations, signifying the significance of this literature in the domain of hydrogel application in the treatment of spinal cord injuries, which may offer fundamental theoretical or experimental approaches for future studies. Ahuja et al. (2017) and Koffler et al. (2019) ranked second and third with 116 and 108 citations, respectively. Recent studies suggest that the utilisation of hydrogels in the management of spinal cord injuries has garnered heightened interest in recent years, particularly with respect to advancements in biocompatibility and therapeutic efficacy. The cluster analysis of the references within the field, as illustrated in Figure 5D, utilises the node size as a quantitative metric for the number of citations, where documents with larger nodes correspond to higher citation counts. Koffler et al. (2019) and Ahuja et al. (2017) are the key papers that have had a significant impact on the field, a result that is consistent with the results of Figure 5A. Figure 5B illustrates the sudden surge of citations for multiple documents, which indicates significant periods of increased attention and innovative breakthroughs in the field during specific time periods. It is notable that the articles by (Jain et al., 2006; Baumann et al., 2010), which have been published in this field for over a decade, have undergone a rapid surge in citations in 2007 and 2011, indicating the seminal role they played in pioneering the application of hydrogels in spinal cord injury research. Recent studies, including those by (Koffler et al., 2019; Fan et al., 2022), have continued to demonstrate a significant upward trend in citations, indicating the growing importance of this literature within the field and its close relation to contemporary treatment strategies and techniques. Figure 5C provides a visual representation of the research contributions of various countries in this field, as well as their collaborative efforts. The data presented in Figure 5C is categorised into two distinct groups: single-country publication (SCP) and multi-country co-publication (MCP). It is evident that China stands as a significant contributor to research in this domain, with a substantially higher number of publications compared to other countries. China’s literature volume is predominantly characterised by single-country publishing, complemented by its engagement in multi-country co-publishing, a practice that is followed by the United States. It is anticipated that these two countries will continue to occupy a prominent position in future research in this field. The sustained growth in international collaborations has the potential to offer enhanced interdisciplinary and geographic support for the utilisation of hydrogel therapy in the management of spinal cord injury. Figures 5E,F illustrate the most frequently cited literature on this subject on a global and local level. Among global citations, the most influential literature includes (Anderson et al., 2016; Banerjee et al., 2009), published in Nature and Biomaterials, respectively. A comparison of the two documents reveals a stark disparity in their global citation counts, with Anderson’s text amassing a staggering 1,299 citations as opposed to Banerjee’s 531. This pronounced discrepancy serves to underscore the substantial academic influence exerted by the former work within the domain of spinal cord injury and hydrogels. The analysis of local citations indicates that the literature of (Koffler et al., 2019; Gupta et al., 2006) dominated the local citation landscape, with 108 and 77 citations, respectively. These documents are of particular significance within their respective research contexts, representing the core research findings in this field. These works provide a foundation for further exploration and advancement in the field, particularly with respect to the development of innovative hydrogel applications in spinal cord injury research.

### 3.5 Keywords and keyword bursts

**FIGURE 6:**
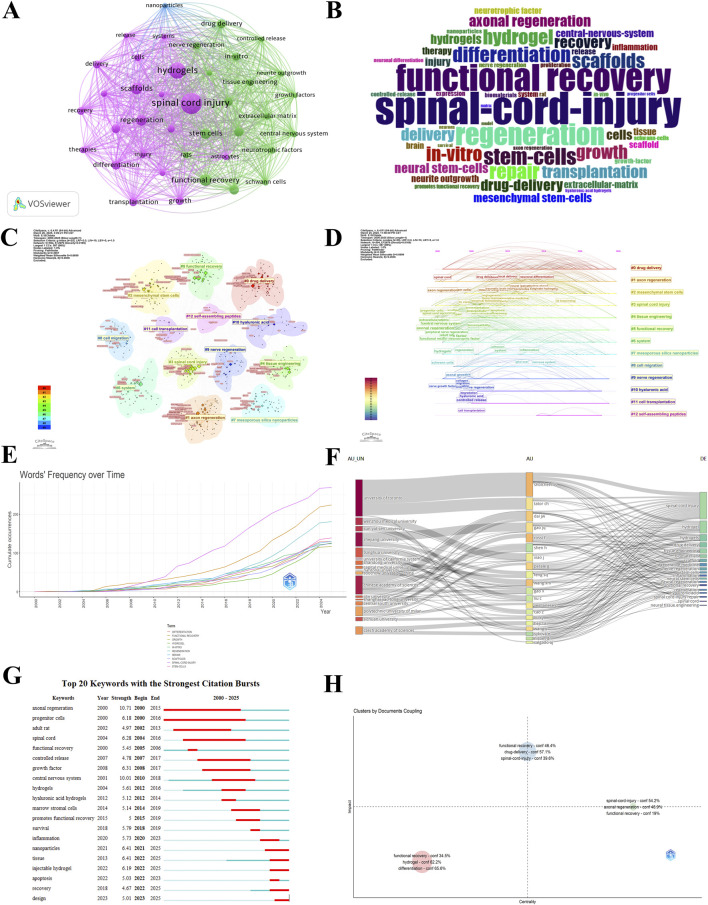
Analysis of keywords and keyword bursts. **(A)** Clusters with more than 50 occurrences of keyword counts. **(B)** Keyword cloud map. **(C)** Cluster visualization of keywords. **(D)** Temporal evolution of the development and interconnection of keyword themes. **(E)** Keywords Frequency Over Time. **(F)** Author and institution collaboration network. **(G)** Top 20 keywords with the strongest citation bursts. **(H)** Relationship between impact and centrality of different themes.

To explore the multidisciplinary nature of SCI research, we conducted a keyword clustering analysis in this field. As shown in Figure 6A, the largest cluster appears in the purple region, centered around the keyword “spinal cord injury.” This cluster is closely associated with terms such as “regeneration”, “functional recovery”, “stem cells” and “hydrogels” indicating that current SCI research is primarily focused on biological mechanisms and therapeutic strategies, especially in tissue repair and regeneration following SCI. The connecting lines between clusters illustrate co-occurrence relationships among keywords. The green cluster, which includes terms like “drug delivery”, “nanoparticles” and “controlled release” highlights a growing research interest in therapeutic delivery systems aimed at enhancing treatment efficacy in SCI.

Figure 6B presents a word cloud of keyword frequencies, where larger font sizes represent higher occurrence rates in SCI-related literature. The keyword “spinal-cord-injury” dominates the cloud, underscoring its central role in the field. Similarly, “functional recovery” and “regeneration” appear prominently, reflecting their significance as primary goals in SCI therapy. Figure 6C shows the co-occurrence network clustering of keywords, providing a structural perspective on the interconnection among research topics. Core topics such as “Axon Regeneration” and “Tissue Engineering” are located in the lower-left quadrant, suggesting that neural tissue engineering forms a fundamental direction in SCI treatment. Meanwhile, “Self-assembling Peptides” and “Cell Transplantation” reside on the periphery, indicating they are emerging frontiers with potential to become future breakthroughs. Figure 6D illustrates the temporal evolution of keyword clusters related to hydrogels in SCI research from 2000 to 2025. Keywords are categorized into 13 clusters, and the timeline reveals the rise and development of various research themes. Notably, “Spinal Cord Injury” and “Functional Recovery” are recurring core targets across multiple clusters, demonstrating that most research directions ultimately converge on functional reconstruction. Emerging technologies such as “System”, “Mesoporous Silica Nanoparticles” and “Self-assembling Peptides” reflect an interdisciplinary trend, encompassing smart hydrogel systems, nanoscale delivery platforms, and self-assembling biomaterials. Overall, this evolutionary map reveals a shift in SCI research focus—from early-stage materials and drug delivery vehicles toward a broader scope including stem cell therapy, tissue engineering, and functional restoration. The role of hydrogels has evolved from serving as simple structural scaffolds to sophisticated systems with bioactive and intelligent response properties. Figure 6E displays the cumulative keyword frequency over time. Terms such as “Stem Cells”, “Spinal Cord Injury”, “Regeneration” and “Functional Recovery” have shown a marked increase since around 2010, indicating growing interest in regenerative medicine and tissue engineering approaches. Keywords like “Hydrogel”, “Scaffolds” and “*In Vitro*” became increasingly prominent after 2015, underscoring their rising importance in SCI repair and regeneration research. Figure 6F maps the relationships among universities, authors, and research themes. The University of Toronto stands out as a major contributor in the hydrogel-based SCI treatment field. Notable researchers such as Shoichet MS and Dai JW appear frequently in the literature, suggesting their pivotal roles in advancing new therapies for SCI repair. Figure 6G highlights the top 20 keywords with the most significant citation bursts from 2000 to 2025. These bursts provide insights into research trends in hydrogel-based SCI treatment. The keyword “Injectable hydrogel” has shown a citation burst since 2022, indicating its potential in localized therapies and targeted drug delivery. Keywords such as “Inflammation” and “Nanoparticles” experienced citation bursts in 2020 and 2021, respectively, reflecting both the significance of immune responses in SCI repair and the growing impact of nanotechnology. The emergence of “Design” as a burst keyword in 2023 may point to innovative therapeutic concepts and hydrogel design strategies. Figure 6H demonstrates the coupling relationship among research themes, where the x-axis represents thematic centrality and the y-axis indicates influence. Clusters such as “Functional Recovery”, “Differentiation” and “Hydrogel” are located in the low-centrality, low-influence quadrant, suggesting that although these topics are strongly interconnected internally, they have not yet exerted broader cross-disciplinary impact. This suggests that hydrogel applications in SCI repair remain under active exploration, with current focus largely on specific mechanisms and experimental outcomes. In contrast, the “Drug delivery”, “Spinal cord injury” and “Functional recovery” cluster occupies the high-centrality, high-influence quadrant, indicating both internal coherence and central positioning within the academic network. The cluster including “Spinal Cord Injury”, “Functional Recovery” and “Axonal Regeneration” lies in the high-centrality but moderate-influence region, suggesting a “connector” role in the scientific structure. Axonal regeneration, as a crucial biological process in SCI repair, is increasingly integrated with hydrogel-based strategies, representing a deeply investigated area with interdisciplinary implications.

## 4 Discussion

This study advances the understanding of hydrogels for spinal cord injury treatment and provides a panoramic view of this domain along with potential future research directions for an extensive spectrum of researchers. To accomplish this, a thorough literature review was executed on the topic in the Web of Science Core collection as of March 2025, yielding 1,015 documents. Research in the field was also statistically scrutinized utilizing bibliometrics.

### 4.1 Key findings

In the realm of bibliometrics, keyword co-occurrences serve as a reflection of prevailing topics within an academic discipline. A timeline representation of these keywords can elucidate the emergence of novel research themes. The frequency of occurrence of a specific keyword is directly proportional to its status as a prominent research topic. By employing techniques such as keyword co-occurrence analysis, clustering, and burst detection, one can identify the current research foci and frontiers in the field of hydrogel applications for spinal cord injury treatment. An examination of the temporal network co-occurrence graph revealed that initial research predominantly focused on progenitor cells and the central nervous system, indicating a primary focus on seed cells centred on nerve regeneration and regenerative rehabilitation as the objective, with hydrogels potentially serving merely as carriers. Keyword burst analysis further revealed that between 2000 and 2010, in addition to spinal cord injury-related themes, research largely focused on topics such as functional recovery and axonal regeneration, corroborating the findings from the temporal network co-occurrence graph. From 2015 to the present, keyword trends beyond spinal cord injury, including inflammation, injectable hydrogel, nanoparticles and recovery, have become popular topics. Research on injectable hydrogels has focused primarily on the functionalization of hydrogel carriers. On the other hand, the theme of inflammation serves as an additional approach to functionalized hydrogels. Cao et al. designed a composite scaffold system consisting of a reactive oxygen species (ROS) filter enveloped by a porous hydrogel loaded with stem cell spheres. This system can rapidly eliminate ROS, diminish oxidative tissue damage, and decelerate secondary injury. Simultaneously, it safeguards stem cells within the scaffold, enhancing their post-implantation survival rate, augmenting the paracrine and neurotrophic effects of plasmacytoid stem cells, and significantly restoring motor and electrophysiological functions in spinal cord-injured rats. This bilayer gel system offers a novel concept for biphasic modulatory treatment of spinal cord injuries (Cao et al., 2023). In a study by Li et al., a bone marrow mesenchymal stem cell (BMSC)-encapsulated ROS-scavenging hydrogel was fabricated by a one-pot synthesis of a thioketal-containing hyperbranched polymer (HBPAK), biocompatible hyaluronic acid-methacrylic anhydride (HA-MA) and peptides encapsulated with cell growth factors and BMSCs. This type of hydrogel has high biocompatibility and can significantly attenuate the oxidative microenvironment *in vitro* and *in vivo* (Li et al., 2023b). An increasing number of studies have focused on functionalized hydrogels that serve to modulate the microenvironment. The production of ROS and inflammation are interrelated processes, with research on inflammation primarily focusing on the use of functionalized hydrogels. This line of inquiry lays a solid groundwork for neural regeneration by optimizing the surrounding microenvironment, which has become a focal point in recent scientific investigations. The exploration of inflammation extends beyond the influence of pharmaceutical agents and the functionality of hydrogels.

### 4.2 Emerging research hotspots in hydrogel-based SCI therapy

In recent years, the study of exosomes has rapidly advanced, as reflected in the surge of related keywords. Fang et al. developed a porous methacrylate gelatine (GelMA) microneedle patch that, when covered with a GelMA hydrogel embedded with bone marrow-derived mesenchymal stem cells (MSCs), facilitated the sustained release of cellular exosomes. This approach reduced the formation of cavernous and scar tissues, promoted angiogenesis, and enhanced the survival of surrounding tissues and axons. Consequently, it led to significant functional recovery in SCI regions. The microneedle patch serves as an efficient platform for sustained cellular exosome delivery, aiding in the treatment of SCI (Fang et al., 2023). Using 3D printing technology, Shang also created personalized GelMA-exosome-siRNA bionic scaffolds that incorporated GelMA hydrogels containing siRNA-loaded MSC-derived exosomes. These scaffolds mitigate inflammatory responses in the spinal cord microenvironment and decrease ECM molecule deposition and scarring. They synergistically enhance axonal growth and nerve fibre connectivity through the PTEN/PI3K/AKT/mTOR pathway, thereby promoting hindlimb motor function after SCI. Encapsulating siRNA-loaded exosomes within individualized 3D-printed bioscaffolds holds great promise for clinical applications in treating spinal cord injuries (Shang et al., 2024). Furthermore, Han chose GelMA hydrogel as a support material and independently constructed a microneedle (MN) array patch (GelMA-MN@3D-exosomes) for SCI repair by loading 3D-cultured MSC-derived exosomes (MSC-Exos). The secreted 3D-Exos, which were enriched in proteins and miRNAs involved in local microenvironmental regulation, demonstrated enhanced neurorestorative abilities. The microneedle patch achieved sustained release of 3D-Exos, effectively reducing inflammation and glial scar formation induced by SCI, representing a promising therapeutic strategy (Han et al., 2022). By immobilizing BMSC-Exos in a conductive hydrogel prepared in this study, we achieved inflammation suppression, NSC recruitment enhancement, and promotion of neuronal and myelin-associated axonal regeneration following SCI treatment. Due to reversible noncovalent binding, the BMSC-derived exosomes within the hydrogels maintained good activity and sustained release, allowing them to accumulate at the site of spinal cord injury in mice. Composite hydrogel implantation promotes NSC recruitment and neuronal and myelinated axon regeneration and inhibits scar-forming gliosis (Fan et al., 2022). Research on nanoparticles has focused primarily on their role as modulatory factors in spinal cord injury and as cytokines in tissue engineering. Recent studies indicate that exosome research is becoming a trend, with a focus on combining this research with multiple themes. The synergistic effect of functionalized materials combined with exosomes or exogenous drugs can regulate the inflammatory microenvironment, resulting in nerve regeneration and functional restoration. This represents not only a current research hotspot but also a new trend in the field.

### 4.3 Challenges in clinical translation

**FIGURE 7:**
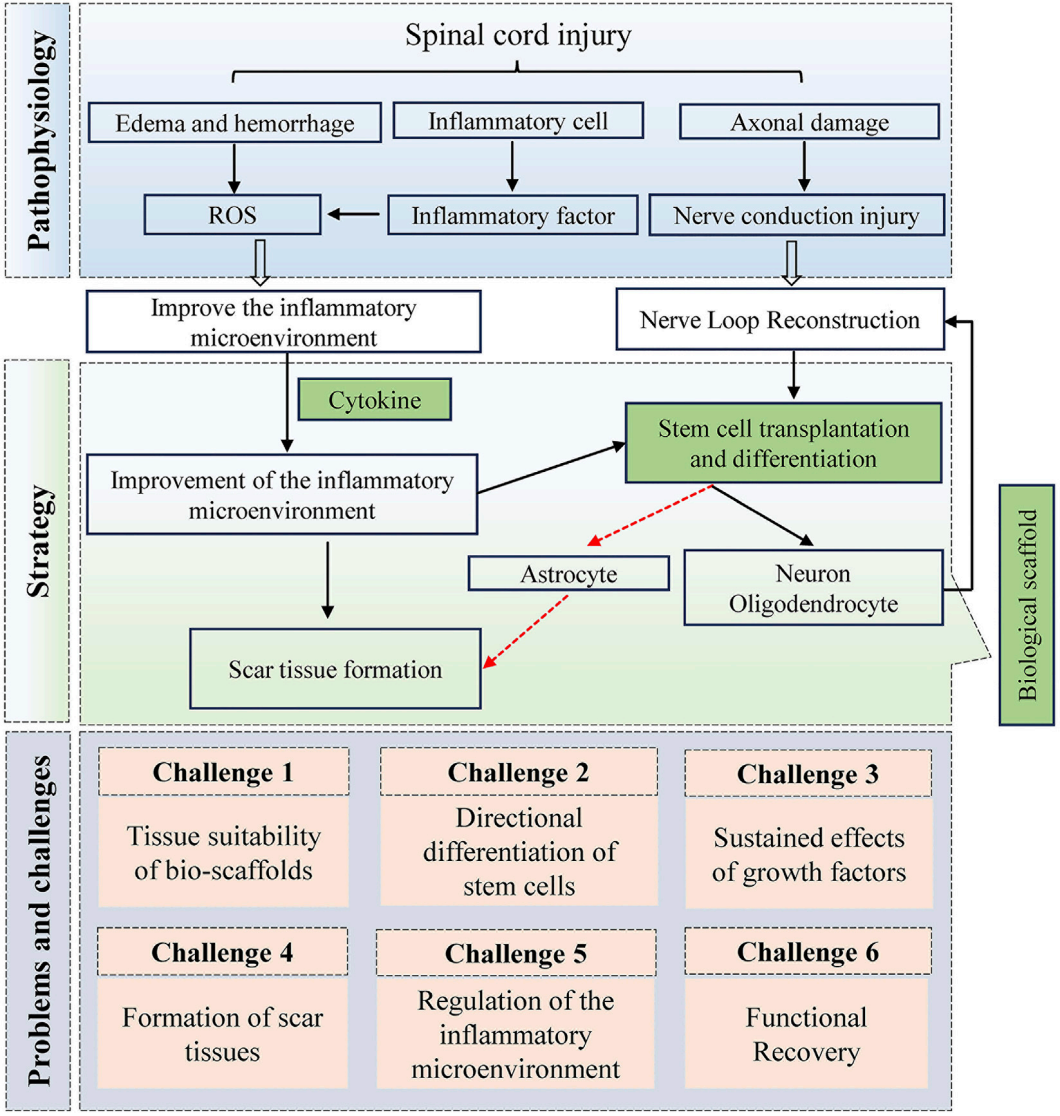
The major treatment strategies and current challenges after spinal cord injury.

In clinical practice, there is currently no effective clinical treatment for nerve regeneration and functional recovery after SCI, making the treatment of SCI a considerable challenge. The complex physiological microenvironment following SCI contributes to the difficulty of nerve repair. Neurons serve as signalling relays, and once damaged, they cannot regenerate. When axonal injury occurs, the intricate inflammatory microenvironment exacerbates demyelinating changes and the demise of damaged neurons. Consequently, endogenous/exogenous seed cells become a crucial factor in nerve regeneration; however, the harsh inflammatory microenvironment accelerates the death of seed cells, and even those that survive tend to differentiate into glial cells rather than neurons (Li et al., 2023a; Qian et al., 2023). Therefore, addressing nerve regeneration necessitates providing a conducive microenvironment for nerve regeneration. A considerable advantage of hydrogel therapy over basic research, such as gene regulation, is its greater potential for clinical translation. Hydrogels are biocompatible, and these bioscaffolds can effectively promote cell differentiation and migration. While focusing on the functionality of the hydrogel, we also need to consider issues related to the sustained delivery of biological factors, as well as the precise release and payload of the drug at specific stages. Improving the microenvironment of spinal cord injury is a prerequisite for treatment, and challenges to be faced in the future include prolonging action efficacy, enhancing the physiological function of seed cells, and compensating for the function of signalling connections in damaged neurons. However, to date, no major breakthroughs involving hydrogel delivery systems for the treatment of human spinal cord injury have been made in clinical trials. This discrepancy between injury models and actual clinical scenarios may be one of the key factors hindering the translation of laboratory studies into clinical applications, making the design of functionalized hydrogels an issue we will focus on in the future. The intricate pathological milieu following spinal cord injury, coupled with existing therapeutic approaches, underscores the formidable challenges impeding future research advancements (Qin et al., 2023; Han et al., 2025; Lv et al., 2022). This complex landscape serves as a pivotal impetus, steering investigators towards an intensified quest for deeper insights within this domain (Figure 7).

### 4.4 Future directions for hydrogel design and application

The analysis of keyword bursts elucidates the importance of the physicochemical properties of hydrogels in treating spinal cord injuries. Hydrogels primarily function as carriers for “seed” cells or bioactive factors. Consequently, enhancing hydrogel properties to optimize spinal cord injury treatment has emerged as a prominent research direction. The rapid advancement of tissue engineering technologies in recent years has led to the development of innovative methods for repairing and regenerating spinal cord injuries. These technologies often utilize biodegradable scaffold materials to replace damaged tissues and introduce exogenous growth factors, thereby providing a conducive biophysical and biochemical environment for cellular growth and effective tissue restoration. Within tissue engineering, the judicious design of scaffold materials, which entails adaptability to the physiological environment and pathological mechanisms of the injured tissue, is crucial for successful tissue repair. Future hydrogel scaffolds should exhibit several key characteristics: (1) appropriate mechanical strength to accommodate the cellular growth environment; (2) adaptable geometry to fill the spinal cord injury defect consistently; (3) resistance to external stresses to prevent scaffold fracture; (4) injectability to support minimally invasive spinal cord injury treatment; (5) a permeable internal structure to facilitate the penetration of growth factors or active ingredients; and (6) selective internal components that enable the acquisition of biologically derived scaffold materials resembling the tissue matrix composition. Ultimately, material characterization determines the therapeutic efficacy in treating spinal cord injury. Hence, addressing the key molecular mechanisms underlying hydrogel therapy for spinal cord injuries through interdisciplinary collaboration, encompassing materials science, cytology, pharmacology, etc., is imperative for enhancing safety and realizing the clinical application of hydrogels. This represents a pressing issue and a focal point in the field.

## 5 Conclusion

The bibliometric analysis systematically reviews 1,015 publications (2000–2025) on hydrogel applications in SCI repair, revealing that China and the United States lead in research output, with Biomaterials as the most prolific journal and Shoichet, Molly S., as the most influential author. Current research hotspots focus on inflammation modulation, nanoparticle-based drug delivery, injectable hydrogels, and functionalized hydrogel systems for microenvironmental regulation, with emerging trends in exosome-loaded hydrogels and 3D bioprinting technologies. The study provides a comprehensive overview of the field’s evolution, highlighting the shift from basic scaffold design to smart responsive hydrogel that integrate biological factors for synergistic therapy. These insights guide researchers in identifying interdisciplinary collaborations and prioritizing translational goals. Future research directions should focus on enhancing the mechanical properties and injectability of hydrogels to accommodate spinal cord defects, developing stimuli-responsive systems for spatiotemporal drug/growth factor release, addressing challenges in clinical translation including bridging preclinical models with human SCI pathophysiology, and exploring exosome-hydrogel conjugates and nanotechnology to modulate immune responses and promote axonal regeneration. As new materials and technologies emerge, the synergies between materials science, pharmacology, and life sciences will be crucial for advancing spinal cord injury treatment.

## 6 Limitations

This study offers valuable insights into hydrogel applications in spinal cord injury research via bibliometric analysis, yet exhibits several analytical limitations. Data are drawn exclusively from the Web of Science Core Collection, risking exclusion of relevant studies from other databases or non-English literature, with inclusion criteria restricted to reviews and articles. The search strategy hinges on predefined keywords, potentially missing emerging terms or interdisciplinary shifts. Furthermore, employment of multiple bibliometric tools introduces algorithmic biases, given the sensitivity of clustering and keyword burst detection to specific software parameters. Incomplete 2025 data and time-dependent metrics additionally constrain temporal analyses. Moreover, while bibliometric analysis effectively identifies research trends and hotspots through keyword and citation data, it may overlook nuanced discussions or small-scale scientific breakthroughs. This approach also fails to fully capture literature quality or impact, as citation counts and publication metrics alone may not reliably gauge research innovativeness or significance.

## Data Availability

The raw data supporting the conclusions of this article will be made available by the authors, without undue reservation.
